# Efficacy and safety of off-label direct oral anticoagulants vs. warfarin for left ventricular thrombus: an inverse probability of treatment weighting analysis

**DOI:** 10.3389/fcvm.2025.1465866

**Published:** 2025-04-28

**Authors:** Taha Al-Maimoony, Khairallah Al-Matari, Abdulhafeedh Al-Habeet, Nouradden Noman Aljaber, Mohamad Al-Marwala, Salah Al-Hashmi

**Affiliations:** ^1^ Department of Cardiology, Faculty of Medicine, Sana’a University, Sana’a, Yemen; ^2^ Department of Internal Medicine, Faculty of Medicine and Health Sciences, Amran University, Amran, Yemen; ^3^ Department of Epidemiology and Biostatistics, Faculty of Medical Sciences, Al-Razi University, Sana’a, Yemen; ^4^ Department of Cardiology, Faculty of Medicine and Health Sciences, Amran University, Amran, Yemen

**Keywords:** direct oral anticoagulants, left ventricular thrombus, warfarin, vitamin K antagonist, inverse probability of treatment weighting analysis

## Abstract

**Objective:**

To evaluate the efficacy and safety of using off-label direct oral anticoagulants (DOACs) compared to warfarin for treating left ventricular (LV) thrombi using inverse probability-of-treatment weighting (IPTW) analysis.

**Methods:**

An observational study of 302 eligible patients with newly diagnosed LV thrombi was conducted at a tertiary referral center from January 2020 to December 2023. Of the 302 patients, 183 received treatment with DOACs, while 119 were treated with warfarin. The primary endpoint was defined as the complete resolution of the thrombus within one month. The secondary endpoints were defined as the complete resolution of the thrombus within six months along with the following events, including minor and major bleeding events, a systemic embolism, transient ischemic attack, stroke, and all-cause mortality. Alongside individual endpoints, a composite endpoint involving ischemic stroke or mortality was also examined.

**Results:**

IPTW estimates suggested that DOACs were significantly more effective than warfarin in resolving LV thrombus within one month (RR: 1.38; 95% CI: 1.14–1.66; *p*-value: <0.001). However, there were no significant differences between the two groups in all secondary endpoints, except that DOACs were significantly associated with a lower incidence of the composite outcome of ischemic stroke and all-cause mortality (RR: 0.96; 95% CI: 0.93–0.99; *p*-value: 0.040). In DOAC subgroup analysis, only rivaroxaban demonstrated earlier and superior resolution of LV thrombus with non-inferior safety when compared to warfarin.

**Conclusions:**

DOACs, specifically rivaroxaban, could be a promising therapeutic alternative for treating LV thrombi. Further research through randomized clinical trials is necessary to confirm our findings.

## 1 Introduction

Left ventricular (LV) thrombus, with an incidence of 6.2%–12.3%, is a serious complication that can occur in LV dysfunction patients typically after nonischemic cardiomyopathy (NICM) or an acute myocardial infarction (AMI) (1–3), leading to an increased risk of systemic embolism and ischemic stroke (4, 5). The pathogenesis of LV thrombus involves a combination of “abnormal blood flow, hypercoagulability, and wall tissue injury” (6). Historically, warfarin was the standard recommendation for treating LV thrombi. Nevertheless, recent evidence from randomized clinical trials (RCTs) and several meta-analyses indicate that direct oral anticoagulants (DOACs) are as effective and safe in treating LV thrombi (7–10). Furthermore, several observational studies showed that patients treated with DOACs after AMI experienced earlier and greater LV thrombus resolution than those treated with warfarin (11, 12).

The European Society of Cardiology (ESC) has recently recommended using DOACs as an alternative anticoagulant treatment to warfarin for 3–6 months to treat patients with LV thrombi (13). DOACs offer a favorable pharmacological and clinical profile, making them an appealing alternative to warfarin. They are easy to administer, do not require regular monitoring of the international normalized ratio (INR), and are free from dietary restrictions. However, whether DOACs could become the first-line strategy is still uncertain. In the present study, we sought to assess the efficacy and safety of using off-label DOACs in treating patients with LV thrombi in a tertiary referral center.

## 2 Materials and methods

### 2.1 Study design and population

An observational study was conducted at the Cardiac Center in Sana'a City, Yemen, from January 2020 to December 2023. Adult patients who had recently been diagnosed with LV thrombus and had been treated with warfarin or DOACs (rivaroxaban or apixaban) were consecutively screened for inclusion. On the other hand, patients with an estimated glomerular filtration rate (eGFR) of less than 15 ml/min, on hemodialysis, with a history of major bleeding, a prosthetic valve, rheumatic mitral stenosis, with valvular atrial fibrillation (AF), those who were undergoing anticoagulation therapy changes during the follow-up period (from warfarin to DOACs or vice versa), those taking potent inhibitors or inducers of cytochrome P450 or non-steroidal anti-inflammatory drugs, or as lactating or pregnant women were excluded.

The study's purpose and process were explained to each patient involved, and written consent forms were obtained. The study was approved by the Cardiac Center's administration and was conducted in accordance with the Declaration of Helsinki.

### 2.2 Assessment of left ventricular thrombus and anticoagulation therapy

The LV thrombus was confirmed by transthoracic echocardiography (TTE) and defined as an echo-dense mass that can be distinguished from endocardium. It has well-defined margins and is next to an aneurysmal, hypokinetic, or akinetic myocardial segment (14). During each TTE, the LV thrombus has to be visible in at least two images during the cardiac cycle. LV ejection fraction (LVEF) was also evaluated. TTE was performed at baseline and within the first, third, and sixth months of follow-up. To ensure accuracy and reduce potential bias, all echocardiograms were performed by a team of blinded echocardiography experts, who were not involved in the clinical care of the patients.

Anticoagulation therapy was administered until LV thrombus resolution was confirmed within a minimum of three months. When LV thrombus resolution could not be proven, anticoagulation therapy was extended for six months or more. The decisions regarding the duration, type, and dose of anticoagulant and concomitant antiplatelet treatment were left to the cardiologist's judgment.

After diagnosing an LV thrombus, patients were orally administered 20 mg of rivaroxaban or 10 mg of apixaban daily, depending on their creatinine clearance. Twenty-eight patients with renal impairment received a modified dose of 15 mg of rivaroxaban, while fourteen patients were given a modified dose of 5 mg of apixaban. Warfarin patients were monitored at the anticoagulation clinic to maintain a target INR range of 2–3. All patients were strictly observed throughout the study period to reach the targeted therapeutic INR levels. All patients in the warfarin group had a time in the therapeutic range of ≥65% throughout the follow-up period.

In the present study, patients treated with DOACs were considered the treatment group (exposure group), while those treated with warfarin were considered the non-treatment group (non-exposure group).

### 2.3 Data collection and control of confounding variables

Confounding by indication occurs when patients receiving a particular therapy inherently differ from those receiving other treatments or no therapy. This poses a significant threat to the validity of nonrandomized comparative treatment efficacy studies (15). To mitigate this bias, the present study encompassed a comprehensive range of variables collected at baseline. This allowed for the effective use of inverse probability-of-treatment weighting (IPTW), which analytically adjusts for indication confounders and then simulates a RCT between warfarin and DOAC users. In clinical practice, patients with LV thrombus are equally likely to receive either DOAC treatment (apixaban or rivaroxaban). This ensures the validity of the DOAC subgroup analysis in our study. Follow-up data was obtained exclusively through outpatient visits.

### 2.4 Study endpoints

While the primary endpoint was defined as the complete resolution of the thrombus within one month, the secondary endpoints were defined as the complete resolution of the thrombus within six months along with the following events including minor and major bleeding events (using BARC criteria) (16), a systemic embolism, transient ischemic attack (TIA), stroke (according to AHA definition) (17), and all-cause mortality within a six-month follow-up. Alongside individual endpoints, a composite endpoint involving ischemic stroke or all-cause mortality was also examined. Persistence of LV thrombi was defined as controversial minor thrombi regressions, stable thrombi sizes, or increased thrombi dimensions at follow-up. At least two skilled and experienced cardiologists validated all clinical events.

All included patients either showed thrombus resolution in their follow-up echocardiogram data or had a minimum six-month follow-up period.

### 2.5 Statistical analyses

Stata/MP software version 17 was used for the statistical analyses. A frequency distribution was initially performed to identify outliers, discrepancies, and missing values. The Kolmogorov–Smirnov test confirmed that all continuous variables were normally distributed. Therefore, the continuous variables were presented as mean ± standard deviation (SD), while categorical variables were represented as numbers with percentages. Depending on the data distribution, the Pearson Chi-square or Fisher's Exact test was used to assess significant differences between categorical variables and anticoagulant groups. Meanwhile, one-way analyses of variance (ANOVAs) were performed to determine significant differences in LVEF across the different anticoagulant groups.

The approach utilized in the study was the inverse probability of treatment (IPTW) analysis. To this end, propensity scores (PSs) were firstly estimated using logistic regression with DOAC treatment as the dependent variable and all confounders and covariates as determinants. To ensure accurate adjustment for covariate imbalances, confounders with absolute standardized difference (ASD) values greater than 0.1 were used in the calculation of the PSs. Subsequently, the average treatment effect (ATE) was estimated by calculating the weighted risk ratio (RR) between DOAC treatment and outcomes. The weights used were 1/PSs for the DOACs group and 1/(1-PSs) for the warfarin group. IPTW generates a simulated database where covariates and confounding variables have no predictive power for the type of anticoagulation therapy. Stabilized weights were then calculated, defined as the inverse of the estimated propensity multiplied by a constant equal to the observed proportion of DOAC users. Using stabilized weights in the pseudo population, the variance of the main effect is appropriately estimated, the type I error rate is kept within acceptable bounds, the weights variability of IPTW is reduced, and, most importantly, the large sample size of the original data is preserved improving the accuracy and reliability of findings (18, 19). Nevertheless, certain stabilized weights were trimmed and reset to 10 (0.1) because they might still be overly large or small. The final weights were calculated by multiplying the treatment weight by the stabilized and trimmed weight (19, 20). Robust standard errors were used to derive the 95% confidence intervals (CIs) for the IPTW estimates (21).

Fractional polynomial plots were employed to assess the associations between continuous variables and the probability of LV thrombus persistence. A *P*-value of <5% was considered a statistically significant difference throughout.

## 3 Results

**Figure 1:**
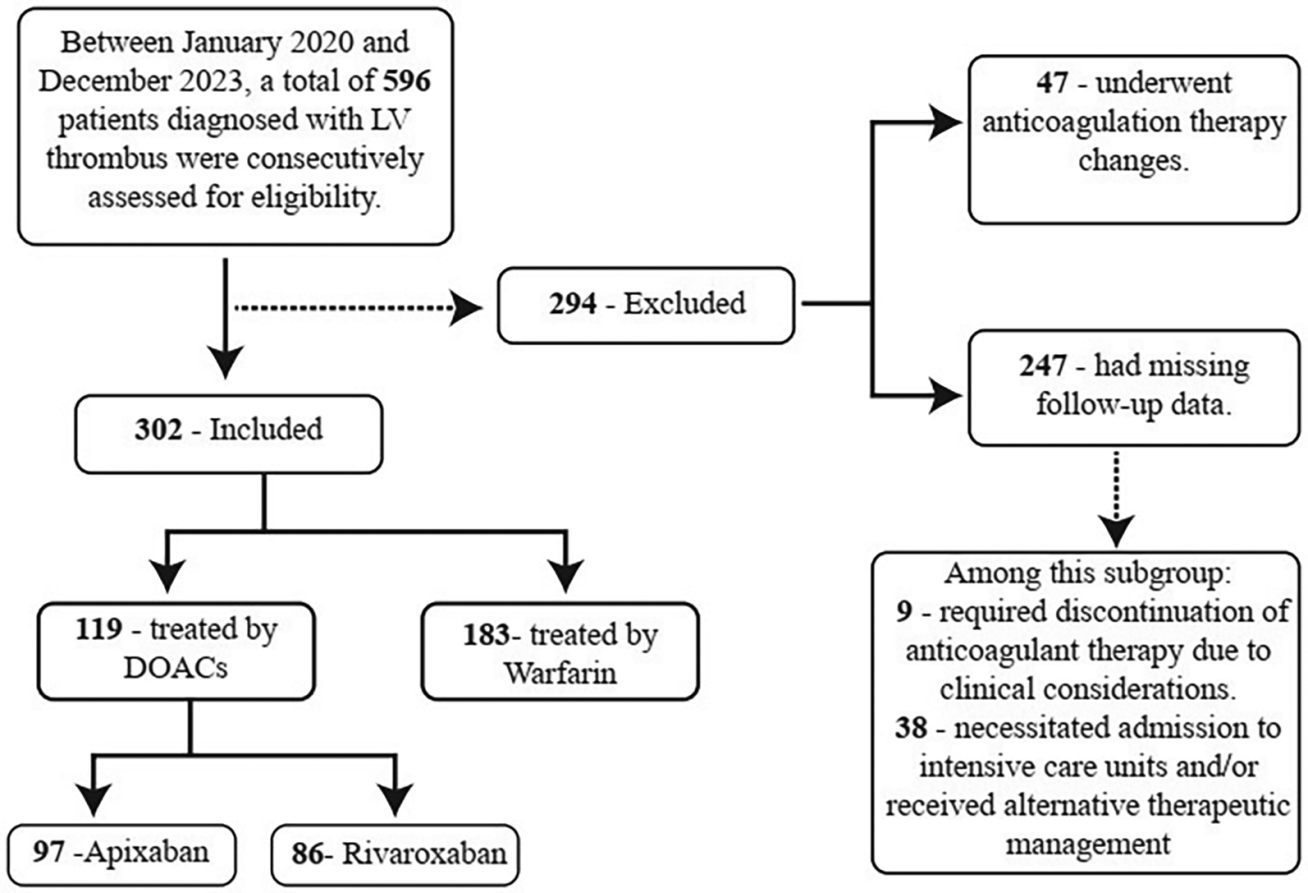
Flowchart of the study population.

During the study period, a total of 596 patients with LV thrombus were screened. Forty-seven patients who underwent anticoagulation therapy changes during the follow-up period were excluded. Furthermore, 247 patients were excluded due to losing their follow-up data. As a result, the final analysis included 302 patients. A detailed summary of the patient selection process and the derivation of the study cohorts is illustrated in Figure 1.

### 3.1 Anticoagulation therapy and baseline characteristics of LV thrombus patients

Of 302 patients, 60.6% (*n* = 183) were treated with DOACs, while 39.4% (*n* = 119) received warfarin treatment. Among the DOAC group, 86 patients were given rivaroxaban, and 97 were given apixaban. The baseline characteristics of patients with LV thrombus in the two treatment groups, along with the ASDs in both the unweighted and weighted cohorts, are presented in Table 1.

**Table 1:** Baseline characteristics of LV thrombus patients in the two anticoagulation groups and absolute standardized differences in entire and weighted cohorts.

Variables	Warfarin (*n* = 119)	DOACs (*n* = 183)	ASD	
			Unweighted cohort	Weighted cohort
Age, mean (SD), Years	56.2 (12.3)	58.4 (11.4)	0.016	0.026
Female gender, *n* (%)	12 (10.1)	16 (8.7)	0.213	0.094
Smoking status, *n* (%)				
Active smokers	9 (7.6)	17 (9.3)	0.479	0.061
Ex-smokers	98 (82.4)	148 (80.9)	0.243	0.002
Non-smokers	12 (10.1)	18 (9.8)	0.238	0.064
Khat chewing, *n* (%)				
Active chewers	25 (21.0)	39 (21.3)	0.448	0.089
Ex-chewers	85 (71.4)	132 (72.1)	0.313	0.049
Non-chewers	9 (7.6)	12 (6.6)	0.204	0.027
Medical history, *n* (%)				
IHD	88 (73.9)	138 (75.4)	0.317	0.033
NICM	9 (7.6)	15 (8.2)	0.121	0.019
Anterior MI	17 (14.3)	35 (19.1)	0.537	0.052
HTN	42 (35.3)	40 (21.9)	0.445	0.002
DM	23 (19.3)	54 (29.5)	0.628	0.005
AF	3 (2.5)	6 (3.3)	0.501	0.018
Prior PCI	50 (42.0)	68 (37.2)	0.308	0.032
Antiplatelet and other medications, *n* (%)				
Aspirin	88 (73.9)	140 (76.5)	0.331	0.018
Clopidogrel	48 (40.3)	72 (39.3)	0.288	0.001
Streptokinase	2 (1.7)	6 (3.3)	1.154	0.006
ARNI	34 (28.6)	106 (57.9)	0.842	0.016
SGLT2i	38 (31.9)	110 (60.1)	0.787	0.012
LVEF, mean (SD), %	34.9 (8.4)	33.7 (9.1)	0.015	0.004
eGFR, mean (SD), (ml/min/1.73 m^2^)	68.0 (28.6)	71.8 (29.2)	0.004	0.007

AF, atrial fibrillation; ARNI, angiotensin receptor/neprilysin inhibitor; ASD, absolute standardized differences; DM, diabetes mellitus; DOAC, direct oral anticoagulants; eGFR, estimated glomerular filtration rate; IHD, ischemic heart disease, LVEF, left ventricular ejection fraction; HTN, hypertension; MI, myocardial infarction; PCI, percutaneous coronary intervention; NICM, nonischemic cardiomyopathy; SGLT2i, sodium-glucose cotransporter-2 inhibitors; SD, standard deviation.

Confounders with ASD values greater than 0.1 were used to calculate PSs.

An ASD value below 0.1 indicates a good balance, with smaller values reflecting better covariate matching. As shown in Table 1, before weighting, nineteen covariates exhibited ASDs greater than 0.1, reflecting a potential imbalance between the treatment groups. However, after applying IPTW, all covariates achieved ASD values below 0.1, indicating successful balancing of measured confounders between the DOAC and warfarin cohorts.

### 3.2 Incidence of primary and secondary endpoints

Table 2 presents the incidence of primary and secondary endpoints in all anticoagulant groups. There were no significant differences in the incidence of primary and secondary endpoints among the anticoagulant groups, except for a notably higher rate of complete thrombus resolution observed in the rivaroxaban and apixaban groups. Patients treated with rivaroxaban achieved significantly higher rates of complete thrombus resolution at one month (40.7%), three months (79.1%), and six months (89.5%) compared to those receiving warfarin (11.7%, 47.9%, and 72.2%). Similarly, patients in the apixaban group exhibited substantial resolution rates of 23.7%, 59.8%, and 79.4% at the same respective follow-up intervals. During the overall follow-up period, the incidence of minor bleeding was 5.8%, 3.1%, and 2.3% in the warfarin, apixaban, and rivaroxaban groups respectively. In the warfarin and apixaban groups, there were 2 (1.6%) and 1 (1.0%) patient with major bleeding and no patients in the rivaroxaban group. Four patients (3.4%) experienced an ischemic stroke in the warfarin group compared to two patients (2.1%) in the apixaban group and one patient (1.2%) in the rivaroxaban group. The incidence of TIA was 2.5%, 3.1%, and 1.2%, while all-cause mortality rates were 5%, 3.1%, and 2.3% in the warfarin, apixaban, and rivaroxaban groups respectively.

**Table 2:** Incidence of primary and secondary endpoints in all anticoagulant groups.

Outcomes	Warfarin (*n* = 119)	Apixaban (*n* = 97)	Rivaroxaban (*n* = 86)	*P*-value
Complete thrombus resolution, *n* (%)				
One-month follow-up (*n* = 72)	14 (11.7)	23 (23.7)	35 (40.7)	**<0.001**
Three-month follow-up (*n* = 183)	57 (47.9)	58 (59.8)	68 (79.1)	**<0.001**
Six-month follow-up (*n* = 240)	86 (72.2)	77 (79.4)	77 (89.5)	**0.010**
Clinical outcomes within six months of follow-up				
LVEF %, mean (SD)	36.0 (9.7)	36.0 (8.0)	36.3 (7.9)	0.963
Minor bleeding, *n* (%)	7 (5.8)	3 (3.1)	2 (2.3)	0.378
Major bleeding, *n* (%)	2 (1.6)	1 (1.0)	0 (0.0)	0.488
Ischemic stroke, *n* (%)	4 (3.4)	2 (2.1)	1 (1.2)	0.575
TIA, *n* (%)	3 (2.5)	3 (3.1)	1 (1.2)	0.675
All-cause mortality, *n* (%)	6 (5.0)	3 (3.1)	2 (2.3)	0.556

DOAC, direct oral anticoagulants; LVEF, left ventricular ejection fraction; TIA, transient ischemic attack. Bold values indicate statistically significant relationships (*P* < 0.05).

### 3.3 Association of DOAC treatment with outcomes

Table 3 presents the ATE estimates of the association of DOAC treatment with all outcomes using IPTW. The estimates suggested that DOACs were significantly more effective than warfarin in resolving thrombus within one month (RR: 1.38; 95% CI: 1.14–1.66; *p*-value: <0.001) and three months (RR: 1.21; 95% CI: 1.11–1.32; *p*-value: 0.001), and were non-inferior within six months resolution (RR: 1.29; 95% CI: 0.95–1.75; *p*-value: 0.104). DOACs were also not significantly different from warfarin in terms of individual outcomes. However, DOACs were significantly related to a lower incidence of the composite outcome of ischemic stroke and all-cause mortality (RR: 0.96; 95% CI: 0.93–0.99; *p*-value: 0.040).

**Table 3:** ATE estimates of the association of DOAC treatment with all outcomes using IPTW.

Outcomes	Warfarin^a^ vs. DOACs		Warfarin^a^ vs. Apixaban	Warfarin^a^ vs. Rivaroxaban
	Unweighted cohort	IPTW cohort		
	RR (95% CI) (*P*-value)			
Complete thrombus resolution				
One-month resolution	1.29 (1.14–1.45) **(<0.001)**	1.38 (1.14–1.66) **(<0.001)**	0.67 (0.44–1.01) (0.056)	1.35 (1.21–1.58) **(<0.001)**
Three-months resolution	1.67 (1.23–2.20) **(<0.001)**	1.21 (1.11–1.32) **(0.001)**	0.94 (0.78–1.12) (0.502)	2.31 (1.49–3.57) **(<0.001)**
Six-months resolution	1.75 (1.12–2.72) **(0.012)**	1.29 (0.95–1.75) (0.104)	1.02 (0.73–1.43) (0.918)	1.75 (1.11–2.75) **(0.011)**
Six-month outcomes				
Minor bleeding	0.96 (0.91–1.02) (0.143)	0.98 (0.95–1.01) (0.260)	0.98 (0.95–1.02) (0.361)	0.98 (0.94–1.02) (0.307)
Major bleeding	0.98 (0.96–1.01) (0.343)	0.98 (0.96–1.00) (0.060)	0.98 (0.96–1.00) (0.239)	NA
Ischemic stroke	0.98 (0.94 (1.02) (0.276)	0.98 (0.95–1.00) (0.084)	0.97 (0.95–1.00) (0.155)	0.98 (0.95–1.00) (0.197)
TIA	0.99 (0.96–1.03) (0.568)	1.00 (0.98–1.02) (0.789)	1.01 (0.98–1.05) (0.260)	0.98 (0.96–1.00) (0.280)
All-cause mortality	0.97 (0.93–1.02) (0.230)	0.98 (0.96–1.01) (0.328)	0.98 (0.95–1.02) (0.358)	0.98 (0.95–1.01) (0.283)
Composite outcome^b^	0.95 (0.89–1.01) (0.092)	0.96 (0.93–0.99) **(0.040)**	0.96 (0.92–1.00) (0.063)	0.96 (0.92–1.00) (0.101)

ATT, average treatment effect in the treated; ATE, average treatment effect; CIs, confidence intervals; DOAC, direct oral anticoagulants; IPTW, inverse probability-of-treatment weighting; NA, not applicable; PSM, propensity score matching; RR, risk ratio; TIA, transient ischemic attack. Bold values indicate statistically significant relationships (*P* < 0.05).

^a^ Reference group.

^b^ Composite outcome includes ischemic stroke and all-cause mortality.

In DOAC subgroup analysis, compared to warfarin, only rivaroxaban demonstrated earlier and superior resolution of LV thrombus [resolution within one month (RR: 1.35; 95% CI: 1.21–1.58; *p*-value: <0.001), three months (RR: 2.31; 95% CI: 1.49–3.57; *p*-value: <0.001), and six months (RR: 1.75; 95% CI: 1.11–2.75; *p*-value: 0.011)] with non-inferior safety. Apixaban showed a comparable anticoagulant effect to warfarin.

### 3.4 Associations of age and LVEF with LV thrombus persistence

**Figure 2:**
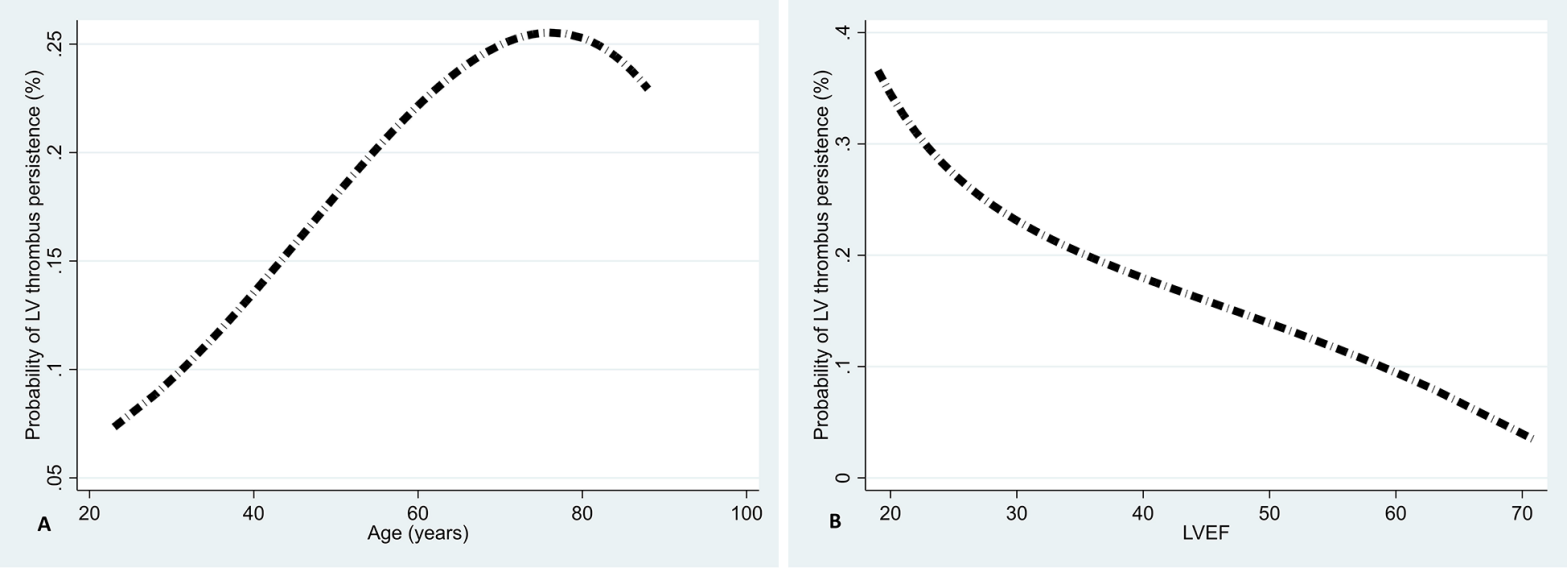
Associations of age **(A)** and LVEF **(B)** with LV thrombus persistence using fractional polynomial plots.

Figures 2A,B illustrate that age is directly associated with LV thrombus persistence (Figure 2A) and LVEF is inversely associated (Figure 2B).

## 4 Discussion

Various factors can distort the treatment-outcome relationship in observational studies. In this situation, PS-based methods, in general, are useful as they provide a neutral weighting mode that gives treatment effects without estimated biases (22). IPTW was used in this study to control for a comprehensive range of confounding variables measured at baseline, preserving the advantage of the relatively large sample size. As a result, treatment of LV thrombi with DOACs was found to significantly reduce the risk of composite outcome of ischemic stroke and all-cause mortality compared to warfarin. Additionally, only rivaroxaban treatment resulted in earlier and superior resolution of LV thrombi.

The main goal of the initial anticoagulation phase is to resolve the thrombus and prevent embolic complications such as stroke and systemic embolization (23, 24). Warfarin is the recommended anticoagulant for treating LV thrombi. Although DOACs were developed recently, evidence supporting their use is still limited.

Reducing the risk of cardioembolic events in the initial months following myocardial infarction (MI) is critical mainly because evidence indicates that most clinical events occur within the first four months after MI (25, 26). Anticoagulation therapy is the cornerstone for preventing these events. An observational study found that treatment with DOACs resulted in a faster resolution of LV thrombi (11). It is important to note that the effectiveness of different DOAC classes and individual agents may vary. All DOACs in our study were oral factor Xa inhibitors. IPTW estimates of the present study revealed that only the rivaroxaban treatment resulted in earlier and superior resolution of thrombi. Several meta-analyses have also indicated that rivaroxaban treatment leads to earlier resolution of thrombi (27), with one study reporting an average duration of rivaroxaban resolution was 30 days (28). The present study finding suggests that treating LV thrombi patients with rivaroxaban could improve their quality of life by shortening treatment duration and reducing associated risks. Our estimates indicate that there is no significant evidence against the similarity of the two anticoagulation strategies in minor bleeding, major bleeding, ischemic stroke, TIA, and mortality, even in DOAC sub-group analysis. However, the composite outcome of ischemic stroke and all-cause mortality showed a lower risk in patients treated with DOACs than those in the warfarin group. The trial sequential analysis confirmed that DOACs effectively promoted LV thrombus resolution and reduced the risk of stroke and all-cause mortality, though no significant benefit was observed for thromboembolism prevention (29). A comprehensive analysis of a recent meta-analysis of twenty-seven studies indicated that, compared to warfarin, DOACs significantly reduced the risk of stroke, all-cause mortality, any bleeding, and major bleeding. Interestingly, its sub-analysis of the RCTs found that these outcomes were no longer significantly different between the two groups. However, only rivaroxaban treatment significantly reduced the risk of stroke (9). An observational study of LV thrombus in post-STEMI patients found that the rate of major bleeding events was lower in the DOAC group compared with the warfarin group, with no difference in the systemic embolism rates (11). The study by Zhou et al. revealed that even after adjusting for smoking and gender, there was no significant difference between the two groups in the resolution of LV thrombi within six months (30). It is noteworthy that in Zhou et al.'s study, the standard dose of DOAC was not fully utilized, and their findings only adjusted for smoking status and gender. This might have made their study more susceptible to the influence of confounding by indication, which could have led to either an overestimation or underestimation of the association between the treatment and the outcome. Conversely, another study by Robinson et al. found that LV thrombi patients treated with apixaban experienced higher rates of systemic embolism and stroke compared to those treated with warfarin (31). Notably, Robinson et al.'s study was retrospective and lacked randomization, which may introduce substantial selection bias. Our findings are more reliable than those of the aforementioned observational studies for several reasons. First, we conducted our study with a relatively large sample size. Second, we meticulously controlled a wide range of baseline variables, contributing to the robustness of IPTW analysis. Third, we observed all outcomes that were essentially free from recall bias exclusively in outpatient clinics, which explains the large number of patients excluded from our study.

Among Yemeni cardiac patients with heart failure or low LVEF, the prevalence of LV thrombus ranges from 6.1%–28.1% (32, 33), indicates a high occurrence of LV thrombus among this population. A low LVEF is a significant and independent risk factor for developing a LV thrombus in dilated cardiomyopathy and AMI patients (34). Even after three months of anticoagulation therapy, a lower LVEF remains an independent predictor of LV thrombus persistence (8). Furthermore, failure to improve LVEF within six months of anticoagulation hinders LV thrombus resolution (35). Our analyses also suggest an inverse association between the overall follow-up LVEF and LV thrombus persistence. Recent evidence from a RCT supports our findings, indicating that patients with impaired LV systolic function, especially those with an LVEF of less than 50%, may face challenges in resolving LV thrombus if their LVEF does not improve. For individuals who have recovered from LV thrombi, the RCT also demonstrated that improving the LVEF is crucial to prevent its recurrence, not just resolving it (35). Therefore, monitoring and enhancing LVEF in managing LV thrombi is essential for promoting successful resolution, improving clinical outcomes, and possibly preventing recurrence. We are intending to extend the study prospectively to investigate the recurrence of LV thrombus and its independent predictors.

## Limitations

Our study may have an important limitation due to its observational design. Specifically, the strategies for anticoagulation therapy were not randomly assigned but were used as part of standard cardiac clinical practice, which likely introduced some bias in the findings. To address this, we conducted IPTW to minimize this bias and achieve a balance between the two anticoagulation therapy strategies based on many factors. However, there may still be some residual bias that could not be eliminated, as IPTW cannot adjust for unmeasured and unknown confounding factors. Another limitation was that a single-center design might limit the generality of our findings. On the positive side, the observational design enabled us to follow a relatively large group of LV thrombi patients using data that is essentially free from recall bias, as it is collected as part of standard cardiac clinical care in our center.

## Conclusions

The present study demonstrated that DOACs were safer and not inferior to warfarin in treating LV thrombi. Only rivaroxaban resulted in an earlier and superior efficacy in resolving LV thrombi than warfarin. Therefore, DOACs, specifically rivaroxaban, could be the preferred anticoagulation for treating LV thrombi. However, further and more comprehensive studies through RCTs are necessary to confirm our findings.

## Data Availability

The raw data supporting the conclusions of this article will be made available by the authors, without undue reservation.
