# Supplementary figures and images for: Efficacy and safety of off-label direct oral anticoagulants vs. warfarin for left ventricular thrombus: an inverse probability of treatment weighting analysis
Source: Front Cardiovasc Med. 2025 Apr 28;12:1465866. doi: 10.3389/fcvm.2025.1465866 (PMC12066775; doi:10.3389/fcvm.2025.1465866)

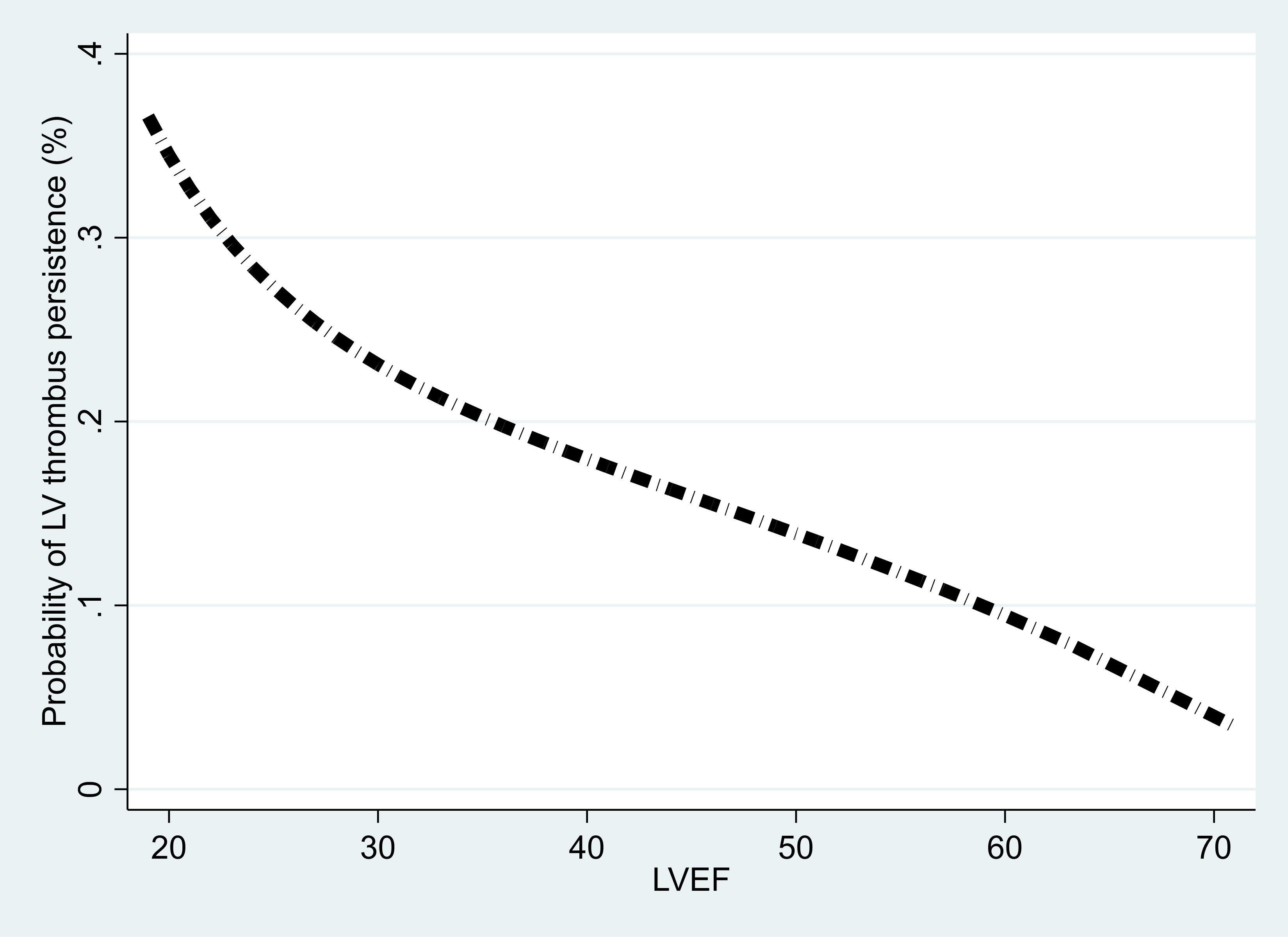

Supplement: Supplementary file 1 [file Image1.png]

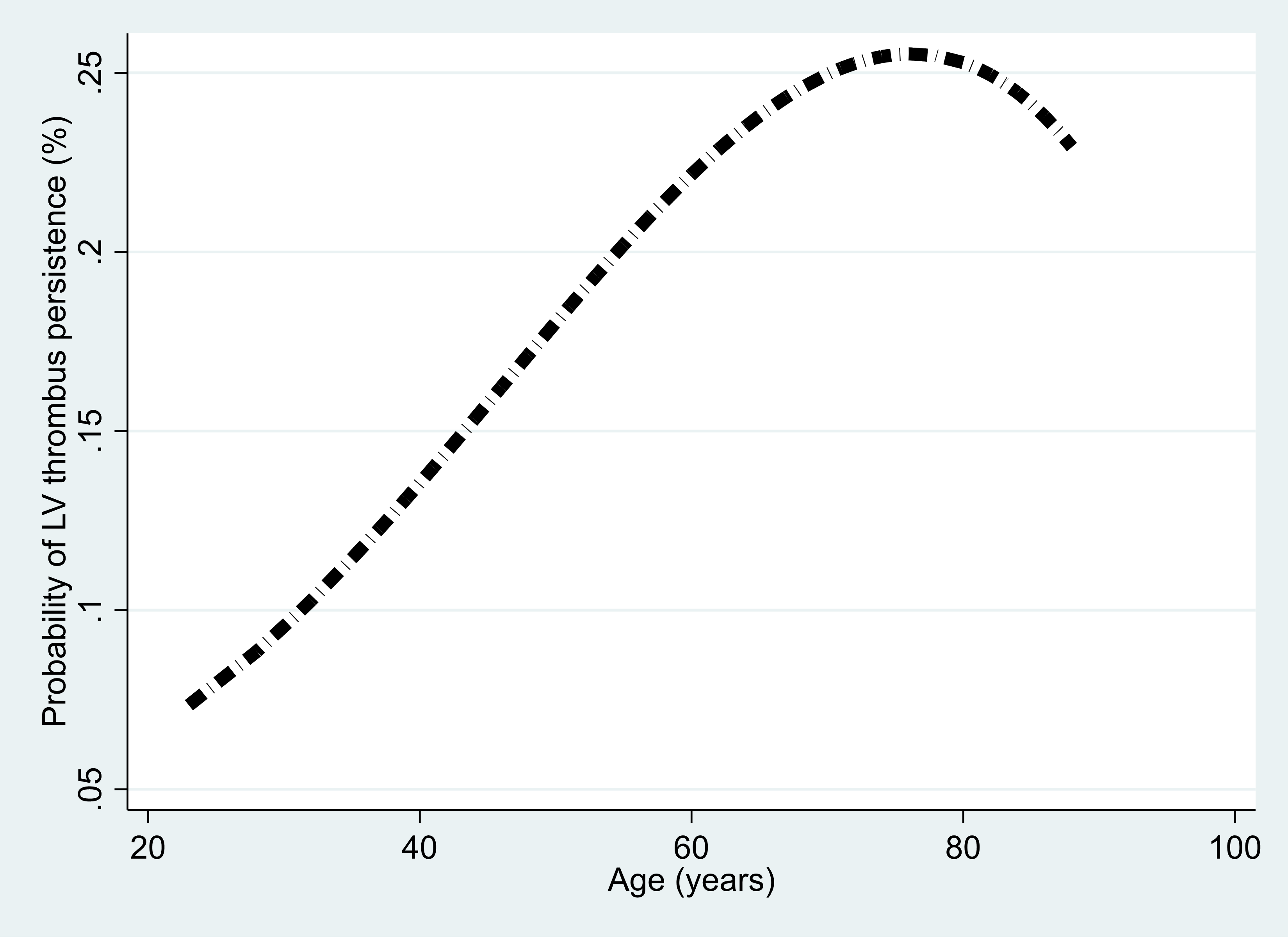

Supplement: Supplementary file 2 [file Image2.png]
